# No considerable changes in papillary thyroid microcarcinoma characteristics over a 30-year time period

**DOI:** 10.1186/s13104-016-2018-2

**Published:** 2016-04-29

**Authors:** Varvara Vlassopoulou, Andromachi Vryonidou, Stavroula A. Paschou, Dimitrios Ioannidis, Angeliki Koletti, Nikolaos Klonaris, Konstantinos Katsoulis, Dimitra Rontogianni, Charalampos Vasilopoulos, Stylianos Tsagarakis, Ioanna Tzavara

**Affiliations:** Department of Endocrinology, Diabetes and Metabolism, “Evangelismos” Hospital, 45-47 Ipsilantou Street, 10676 Athens, Greece; Department of Endocrinology, Diabetes and Metabolism, Hellenic Red Cross Hospital, Athens, Greece; Department of Endocrinology, Diabetes and Metabolism, “Amalia Fleming” Hospital, Athens, Greece; Department of Pathology, “Evangelismos” Hospital, Athens, Greece

**Keywords:** Thyroid, Cancer, Microcarcinoma, Papillary

## Abstract

**Background:**

The prevalence of papillary thyroid microcarcinoma (PTMC) is continuously increasing but its clinical significance and management is still debatable. The aim of this study was to investigate possible changes in the clinical presentation, tumor characteristics, treatment modalities and long-term outcome during the last three decades in patients with PTMC.

**Methods:**

We studied 335 patients with PTMC who were followed up for at least 5 years, from 1982 to 2015, and treated in accordance with the current literature or guidelines at each time-period. Patients were classified according to year of diagnosis into two time periods, TP1 from 1982–2000 and TP2 from 2001–2010.

**Results:**

The mean follow-up of the whole cohort was 10.6 ± 5.3 (median 9) years. No change was noted in the mean age at diagnosis or the female to male ratio during the two time periods. In regard to tumor characteristics, multifocality and non-encapsulated follicular variant of PTMC was more often present while classic PTMC was less common in patients in the TP2, compared to patients in the TP1 (p = 0.007, p < 0.001 and p = 0.043 respectively). The prevalence of incidental PTMC was high but similar in both time periods (84.6 vs 80 %, p = 0.286). The majority of patients in TP2 underwent a total or near total thyroidectomy compared to patients in TP1 (91.7 vs 80 %, p = 0.001). However, more patients underwent thyroidectomy for toxic multinodular disease and less for Graves’ disease during TP1 compared to patients in the TP2 (p = 0.02 and 0.043 respectively). A significant percentage of patients underwent adjuvant radioiodine ablation, yet no difference was found between the two time periods (73.8 vs 79.5 %, p = 0.228). The rate of persistence was very low and not significant (3.1 vs 6.6 %, p = 0.165), while disease recurrence was observed in only 2 (0.6 %) patients, one from each time period.

**Conclusions:**

We did not observe any important changes regarding the clinical presentation or tumor characteristics of PTMCs during a 30-year period. With applied interventions a favorable course was confirmed in the majority of patients without differences in recurrence or persistence during the last three decades.

## Background

During the last decades, papillary thyroid cancer (PTC) has shown the largest increase in incidence amongst all human solid cancers worldwide [1–4]. This increase is mostly the result of identification of smaller thyroid tumors due to the routine use of high-resolution thyroid ultrasonography (US) and imaging for non-thyroid related conditions, as well as the application of total or near total thyroidectomy in surgery [5, 6].

Papillary thyroid carcinomas equal to or less than 1 cm are classified as papillary thyroid microcarcinomas (PTMCs) [7]. According to autopsy studies they seem to be frequent and often unnoticed during the entire lifespan [3, 4]. Since PTC presents itself an overall excellent prognosis with a 5-year survival of 97 % [8, 9] aggressive management of such small thyroid malignancies is rather debatable. In fact recent guidelines based mainly on retrospective studies recommend a non-aggressive treatment for PTMC similar to that of any other benign thyroid nodule and highlight no need for completion thyroidectomy, prophylactic lymph node dissection, radioiodine ablation and suppressive thyroxine treatment [10, 11].

On the other hand, several studies have identified lymph node metastases at presentation, loco-regional recurrences during follow-up and rarely distant metastases and even cancer-related deaths. These latter studies report several risk factors for recurrence and emphasize both the importance of early identification of PTMCs and their aggressive management as in non-PTMCs [12–18]. However, most studies on PTMCs have a relatively short follow-up. As recurrences may occur later it is obvious that there is a need for longer follow-up studies. In the present study we retrospectively investigated the outcome of 335 patients with PTMCs followed up regularly for a minimum of 5 years and up to 33 years. Moreover we investigated possible changes in the clinical presentation, tumor characteristics and outcome of PTMCs presented during a 30-year time period in Greece performing a comparison between earlier and more recent decades.

## Methods

During a 30-year time period, from 1982 to 2010, a total number of 523 patients with PTMC attended the Departments of Endocrinology, Diabetes & Metabolism at “Evangelismos” Hospital, the Hellenic Red Cross Hospital and “Amalia Fleming” Hospital in Athens, Greece. Of them, 335 patients have been followed up for at least 5 years and were included in the study.

Research has been approved by the Ethics Committees of the “Evangelismos” Hospital, Hellenic Red Cross Hospital and “Amalia Fleming” Hospital in Athens, Greece and all clinical investigations have been conducted according to the principles expressed in the declaration of Helsinki.

In order to examine possible changes in the clinical presentation, tumor characteristics, treatment modalities and long-term outcome, patients were divided into two time periods from 1982–2000 and from 2001–2010. Division was based upon the year of diagnosis and change in patients’ approach with the introduction of recombinant TSH in the follow-up of low risk PTC [8]. Written informed consent was obtained from all participants.

Routine use of thyroid ultrasonography (US) was established in Greece since 1995 and 48/130 (36.9 %) patients underwent this examination during the first time period, while 205/205 (100 %) patients during the second time period. Regarding fine-needle aspiration, its routine use was established in Greece since 1999 and 38/130 (29.2 %) patients underwent pre-operatively this examination during the first period, while 156/205 (76.1 %) patients during the second time period. Radioisotope scan prior to surgery was performed in 19/130 (14.6 %) patients during the first time period, while in 15/205 (7.3 %) patients during the second time period. The surgical policy of our endocrine departments was lobectomy for toxic adenomas and total or near total thyroidectomy for the rest cases. The pathological examination of the thyroid specimens was based on the criteria of gross examination that have been established by Nikiforov [19]. The pathological blocks were corresponded one in every 5 gr of the thyroid specimens.

Follow up was conducted at 6–12 months after initial treatment and yearly thereafter or more often, depending on the clinical course. At each visit, all patients underwent physical examination, imaging studies (cervical ultrasonography or I^131^ whole body scan) and measurement of serum thyroglobulin levels with concurrent thyroglobulin antibodies assessment, off-thyroxine therapy. From 2001 onwards, assessment of disease outcome was mainly performed with cervical ultrasonography and thyroglobulin stimulation with recombinant human thyrotropin (Thyrogen, Genzyme Copr., Cambridge, MA) [10, 11].

Thyroglobulin was measured with an IRMA method (Sorin, Biomedica Saluggia, Italy) with a detection limit of 3 mg/l, which from July 1995 was modified to 1 mg/l. Patients with elevated basal thyroglobulin levels (>3 mg/l until 1995 and >1 mg/l thereafter) or stimulated (>3 mg/l after 2001) were classified as having persistent or recurrent disease even if other tests (including whole body scan) were negative, as previously described [20]. However thyroglobulin values just over the detection limit were not considered as indicative for active disease, unless confirmed at the same time by imaging or biopsy or by a repeatedly elevated thyroglobulin value. Thyroglobulin values were not used in the follow up of patients with positive thyroglobulin antibodies. TSH was measured with IRMA (International CIS, Gif-sur-Yvette, France) during 1980s and sensitive IRMA method (Henning, Berlin, GMBH) with analytical sensitivity of 0.03 mU/ml, thereafter.

New evidence of disease occurring more than 1 year after initial diagnosis was classified as cancer recurrence. Cancer was classified as persistent if residual invasive or metastatic disease persisted despite therapeutic interventions more than 1 year after initial diagnosis.

### Statistical analysis

Results are presented as mean ± SD for continuous variables and as absolute numbers or percentages for categorical variables. Distribution of continuous parameters was tested by Kolmogorov–Smirnov Test. Differences in continuous variables between groups were tested using Independent T Test or Mann–Whitney U Test, as appropriate. Differences in categorical variables between groups were tested using χ^2^ test with Yates correction and Fisher’s exact test when needed. All statistical analyses were performed using the Statistical Package for Social Sciences (SPSS 16.0, Inc, Chicago, IL, USA). A p value of < 0.05 was considered statistically significant.

## Results

The clinical and histopathological characteristics of the whole cohort are presented in Table 1.

**Table 1:** Clinical and histopathological characteristics of the whole cohort

Number of patients	335
Age at diagnosis	48.9 ± 12.6 (range 16–81) years
Gender	264F (78.8 %)
	71 M (21.2 %)
Hyperthyroidism	46/335 (13.7 %)
Graves	21/46 (45.7 %)
Hashimoto	47/335 (14 %)
Family history	5/335 (1.5 %)
Singlenodular	87/335 (26 %)
Multinodular	248/335 (74 %)
Incidental	274 (81.8 %)
Non-incidental	61 (18.2 %)
Unifocal	215/335 (64.2 %)
Multifocal	120/335 (35.8 %)
<or = 5 mm	109/215 (50.7 %)
>5 mm (of unifocal cases)	106/215 (49.3 %)
Metastases	0/335 (0 %)
Capsule involvement	68/335 (20.3 %)
T > 1 and/or N > 0	36/335 (10.7 %)
Histological types	170/335 (50.7 %) CPC
	61/335 (18.2 %) EFVC
	58/335 (17.3 %) N-EFVC
	41/335 (12.3 %) SVC
	5/335 (1.5 %) T-CVC
Total or near total thyroidectomy	292/335 (87.2 %)
I^131^	259/335 (77.3 %)
Follow up	10.6 ± 5.3 (median 9, range 5–33) years
Recurrence	2/335 (0.6 %)
Persistence	13/335 (3.9 %)
Disease free	320/335 (95.5 %)

*CPC* classic papillary, *EFVC* encapsulated follicular variant, *N-EFVC* non-encapsulated follicular variant, *SVC* sclerosing variant, *T-CVC* tall-cell variant

Patients were followed up for 10.6 ± 5.3 (median 9, range 5–33) years. Mean age at diagnosis was 48.9 ± 12.6 (range 16–81) years and the majority of them were females (264, 78.8 %).

Two hundred ninety two (87.2 %) patients underwent a total or near total thyroidectomy (24 h uptake <5 %) and 43 (12.8 %) patients an incomplete thyroidectomy (24 h uptake >5 %) of whom 13 were submitted to further completion surgery. Hyperthyroidism was present at diagnosis in 46 (13.7 %) of these patients, of whom 21 were diagnosed with Graves’ disease. In 47 patients (14 %) autoimmune thyroiditis was present. Family history was positive only in five patients (1.5 %). Eighty-seven patients (26 %) presented with single nodular and 248 (74 %) with multinodular disease. From the patients who presented with a single nodule, 26 of them had a FNA/LN positive, 15 had hyperthyroidism (five with Graves’ disease and 10 with Toxic Adenoma) and the remaining 36 had an incidental PTMC inside a prominent benign nodule.

PTMC was discovered incidentally in 274 (81.8 %) patients undergoing surgery for other pathology non-related to thyroid malignancy, while 61 (18.2 %) patients were operated for suspicious FNA or positive lymph nodes. Unifocal PTMC was detected in 215 (64.2 %) patients with 109 of them (50.7 %) having a focus of 1–5 mm and the remaining 106 (49.3 %) a focus of 6-10 mm. Multifocal disease was detected in 120 (35.8 %) patients. None of these patients had distant metastases. However loco-regional extension and lymph-node involvement (T > 1 and/or N > 0) were detected in 36 patients (10.7 %); 22 with unifocal and 14 with multifocal disease (p > 0.05). Of these 22 cases with unifocal disease and loco-regional extension/lymph-node involvement, 10 had a lesion of 1-5 mm and 12 a lesion of 6-10 mm (p > 0.05). Capsule involvement was present in 68 (20.3 %) of whom 43 had unifocal and 25 multifocal disease (p > 0.05). Amongst the 43 patients with unifocal disease and capsule involvement 12 had a lesion of 1–5 mm and 31 a lesion of 6–10 mm (p < 0.001).

The histological subtypes of PTMC were as follows: 170 (50.7 %) classic papillary (CPC), 61 (18.2 %) encapsulated follicular variant (EFVC), 58 (17.3 %) non-encapsulated follicular variant (N-EFVC), 41 (12.3 %) sclerosing variant (SVC) and 5 (1.5 %) tall-cell variant (T-CVC).

Two hundred fifty nine (77.3 %) patients of the whole cohort underwent adjuvant radioiodine ablation due to worrisome histologic subtypes, multifocal disease, thyroid capsule invasion, lymph node involvement or thyroid remnants. The mean follow-up of the whole cohort was 10.6 ± 5.3 (median 9, range 5–33) years. During follow up disease recurrence was observed in only 2 (0.6 %) patients, six and 20 years after initial diagnosis and remission. Both of them had an incidental PTMC, classified as T1N0 and a negative family history. One of them had undergone adjuvant radioiodine ablation. The recurrence was in the thyroid bed alone.

Thirteen of 335 (3.9 %) patients had persistent disease at the end of the follow up. The mean age at diagnosis of these patients was 55.3 ± 14.2 (range 37–81) years and seven of them were men. Four of them presented with hyperthyroidism, three of whom had Graves’ disease. One had Hashimoto disease. None of them had a positive family history. Only one presented with a single nodule. Five of them underwent surgery for suspicious FNA or positive lymph nodes and in the other eight patients PTMC was discovered incidentally. Seven of these 13 patients had unifocal disease of whom only two had a focus greater than 5 mm. Capsule involvement was present in three of these patients, 1 of them was T > 1 and five of them N > 0 (4 CPC, 1 SVC). The histological subtypes in these patients were: 10 CPC, 2 SVC and 1 N-EFVC. All these 13 patients underwent thyroid remnant ablation and 8 of them received further radioiodine therapy (250–420 mCi). The remaining five patients did not undergo further radioiodine treatment due to different causes (three lost to follow-up, one with relapsing chronic pancreatitis and one unwilling to stay in the special room).

We found no statistically significant difference in persistence (0.4 vs 1.3 %, p = 0.354) or recurrence (3.1 vs 6.6 %, p = 0.165) events between the radioiodine ablated and non-ablated patients, respectively. The remaining 320/335 (95.5 %) patients were free of disease.

### Comparison between PTMC patients presented into two time periods

Patients were classified into two time-period groups according to the year of diagnosis: time period 1, 1982–2000 (n = 130) and time period 2, 2001–2010 (n = 205). Comparisons of clinical and histopathological characteristics between the two groups are presented in Table 2.

**Table 2:** Comparison of the clinical and histopathological characteristics between the time periods 1982–2000 and 2001–2010

	1982–2000	2001–2010	*P* value
Number of patients	130	205	
Age at diagnosis	47.8 ± 11.8 (range 20–73) years	49.2 ± 13.1 (range 16–81) years	0.321
Gender	108F (83.1 %)	156F (76.1 %)	0.128
	22 M (16.9 %)	49 M (23.9 %)	
Hyperthyroidism	24/130 (19.2 %)	22/205 (10.2 %)	*0.033*
Graves	7/24 (32 %)	14/22 (61.9 %)	*0.019*
Toxic Adenoma	4/24 (16.6 %)	2/22 (9.1 %)	0.370
Toxic MNG	13/24 (54.2 %)	6/22 (27.3 %)	0.064
Hashimoto	15/130 (11.5 %)	32/205 (15.6 %)	0.296
Family history	3/130 (2.3 %)	2/205 (1 %)	0.327
Singlenodular	37/130 (28.5 %)	50/205 (24.4 %)	0.408
Multinodular	93/130 (71.5 %)	155/205 (75.6 %)	
Incidental	110/130 (84.6 %)	164/205 (80 %)	0.286
Non-incidental	20/130 (15.4 %)	41/205 (20 %)	
Unifocal	95/130 (73.1 %)	120/205 (58.5 %)	*0.007*
Multifocal	35/130 (26.9 %)	85/205 (41.5 %)	
< or = 5 mm	46/95 (48.4 %)	63/120 (52.5 %)	0.552
>5 mm (of unifocal cases)	49/95 (51.6 %)	57/120 (47.5 %)	
Metastases	0/130 (0 %)	0/205 (0 %)	>0.999
Capsule involvement	23/130 (17.7 %)	45/205 (22 %)	0.345
T > 1 and/or N > 0	15/130 (11.5 %)	21/205 (10.2 %)	0.709
Histological types	75/130 (57.7 %) CPC	95/205 (46.3 %)	*0.043*
	25/130 (19.2 %) EFVC	36/205 (17.6 %)	0.699
	11/130 (8.5 %) N-EFVC	47/205 (22.9 %)	<*0.001*
	17/130 (13.1 %) SVC	24/205 (11.7 %)	0.709
	2/130 (1.5 %) T-CVC	3/205 (1.5 %)	0.956
Total or near total thyroidectomy	104/130 (80 %)	188/205 (91.7 %)	*0.001*
^131^I	96/130 (73.8 %)	163/205 (79.5 %)	0.228
Follow up	13.9 ± 6.6 (range 5–33) years	8.5 ± 2.7 (range 5–14) years	<*0.001*
Recurrence	1/130 (0.8 %)	1/205 (0.5 %)	0.745
Persistence	3/130 (2.3 %)	10/205 (4.9 %)	0.235
Disease free	126/130 (96.9 %)	194/205 (94.6 %)	0.324

No change was noted in the mean age at diagnosis or the female to male ratio during the two time periods (p > 0.05). In regard to tumor characteristics, multifocality and N-EFVC was more often present while classic PTMC was less common in patients in the TP2, compared to patients in the TP1 (p = 0.007, p < 0.001 and p = 0.043 respectively).

The prevalence of incidental PTMC was high but similar in the two time periods (84.6 vs 80 %, p = 0.286). The majority of patients in TP2 underwent a total or near total thyroidectomy compared to patients in TP1 (91.7 vs 80 %, p = 0.001). However, more patients underwent thyroidectomy for toxic multinodular disease and less for Graves’ disease during TP1 compared to patients in the TP2 (p = 0.02 and 0.043 respectively).

A significant percentage of patients underwent adjuvant radioiodine ablation, yet no difference was found between the two time periods (73.8 vs 79.5 %, p = 0.228). The rate of persistence was very low and not significant (3.1 vs 6.6 %, p = 0.165), while disease recurrence was observed in only 2 (0.6 %) patients, one from each time period.

### Comparison between incidental and non-incidental PTMCs

The clinical and histopathological characteristics of patients with incidental and non-incidental PTMCs are presented in Table 3.

**Table 3:** Comparison of the clinical and histopathological characteristics between incidental and non-incidental PTMCs

	Incidental PTMCs	Non-incidental PTMCs	*P* value
Number of patients	274	61	
Age at diagnosis	49.1 ± 12.5 (range 16 – 76) years	47.9 ± 13.1 (range 18–81) years	0.485
Gender	226F (82.5 %)	38F (62.3 %)	<*0.001*
	48 M (17.5 %)	23 M (37.7 %)	
Hyperthyroidism	46/228 (20.2 %)	0/61 (0 %)	<*0.001*
Graves	21/46 (45.7 %)	0/0	–
Hashimoto	42/274 (15.3 %)	5/61 (8.2 %)	0.147
Family History	4/274 (1.5 %)	1/61 (1.6 %)	0.917
Singlenodular	65/274 (23.7 %)	22/61 (36.1 %)	*0.047*
Multinodular	209/274 (76.3 %)	39/61 (63.9 %)	
Unifocal	179/274 (65.3 %)	36/61 (59 %)	0.352
Multifocal	95/274 (34.7 %)	25/61 (40.1 %)	
< or = 5 mm	96/179(53.6 %)	13/36 (36.1 %)	0.055
> 5 mm (of unifocal cases)	83/179 (46.4 %)	23/36 (63.9 %)	
Metastases	0/274 (0 %)	0/61 (0 %)	>0.999
Capsule involvement	21/274 (7.7 %)	47/61 (77 %)	<*0.001*
T > 1 and/or N > 0	17/274 (6.2 %)	19/61 (31.1 %)	<*0.001*
Histological types	145/274 (52.9 %) CPC	25/61 (41 %)	0.091
	53/274 (19.4 %) EFVC	8/61 (13.1 %)	0.254
	36/274 (13.1 %) N-EFVC	22/61 (36 %)	<*0.001*
	37/274 (13.5 %) SVC	4/61 (6.6 %)	0.134
	3/274 (1.1 %) T-CVC	2/61 (3.3 %)	0.203
Total or near total thyroidectomy	240/274 (87.5 %)	52/61 (85.2 %)	0.620
^131^I	202/274 (73.7 %)	57/61 (93.4 %)	<*0.001*
Follow up	10.9 ± 5.4 (range 5–33) years	9.1 ± 4.3 (range 5–27.5) years	*0.005*
Recurrence	2/274 (0.7 %)	0/61 (0 %)	0.503
Persistence	8/274 (3 %)	5/61 (8.2 %)	0.054
Disease free	264/274 (96.3 %)	56/61 (91.8 %)	0.120

Mean age at diagnosis was similar for patients with incidental and non-incidental PTMCs. There was a statistically significant higher proportion of women in the cases of incidental PTMCs (82.5 vs 62.3 %, p < 0.001). The prevalence of hyperthyroidism at diagnosis was higher in patients of the incidental PTMCs group (20.2 vs 0 %, p = <000.1) as none of the patients who underwent FNA or had positive lymph nodes had hyperthyroidism. Hashimoto thyroiditis was present at similar percentages in the two groups. Family history was positive at the same low percentages in both groups. The proportion of patients presenting with a single nodule was statistically significantly higher in the group of patients with non-incidental PTMCs, while the patients who underwent surgery for other pathology non-related to thyroid malignancy had multinodular disease at higher proportion (p = 0.047).

Unifocal disease as well as the size of the focus did not differ between patients with incidental and non-incidental PTMCs. Loco-regional extension and/or lymph-node involvement (T > 1 and/or N > 0) were detected in a statistically significantly higher percentage in patients with non-incidental PTMCs (6.2 % vs 31.1 %, p < 0.001). Capsule involvement was also present in a much higher proportion in the group of non-incidental PTMCs (7.7 vs 77 %, p < 0.001). As far as the histological subtypes of PTMC are concerned we found a significantly higher frequency of the N-EFVC subtype in patients from the group of non-incidental PTMCs (13.1 vs 36 %, p < 0.001). We did not find any other differences regarding the rest histological subtypes.

The type of surgery did not differ between the two groups of patients. However, a statistically significantly higher proportion of patients with non-incidental PTMCS underwent adjuvant radioiodine ablation (73.7 vs 93.4 %, p < 0.001). Disease recurrence was observed in two patients with incidental PTMCs. Persistent disease was more frequent in patients with non-incidental PTMCs but without achieving statistical significance (3 vs 8.2 %, p = 0.054). The vast majority of patients with incidental and non-incidental PTMCs were disease free without any statistically significant difference (96.3 vs 91.8 %, p = 0.120).

## Discussion

In the present study we confirmed the benign course of PTMC after a detailed and extensive follow-up period ranging from 5 up to 33 years. Only a minute proportion of patients (0.6 %) presented with a recurrence. However, a small percentage (3.9 %) of patients had persistent disease more frequently in those with non-incidental PTMCs but without achieving statistical significance. In this cohort we did not find any remarkable differences in the presentation, clinical characteristics, histology and outcome of the most recently detected cases compared to those detected in previous decades. Also we did not find any significant difference in persistence or recurrence between the radioiodine ablated and non-ablated patients.

Our results are in agreement with most studies, which showed that PTMCs have excellent prognosis with a mortality of about 1 % and recurrence or persistence of only 1.4–10.5 % [12, 21–29]. Furthermore the study by Ito et al. [30] who prospectively followed up a large group of patients with PTMCs without any intervention did not show poorer prognosis for these patients. The findings of our study gain importance in the light of the detailed and extensive follow-up from 5 to 33 years. Furthermore the lack of differences in persistence or recurrence between the radioiodine ablated and non-ablated is in agreement with previous studies [12, 22–30] and recent guidelines that suggest no need for completion thyroidectomies, lymph node dissections, radioiodine ablation and suppressive thyroxine treatment for PTMCs [10, 11, 31].

Nevertheless, the potential aggressiveness of PTMCs demonstrated by some studies [12–18] has raised the question of identifying possible risk factors for mortality and recurrence, which could help clinicians in deciding which patients need to be treated as if they had any other worrisome papillary thyroid carcinoma. These factors are described to be mostly the size of the tumor, the capsule involvement and invasiveness, the presence of lymph node metastases and multifocality [32, 33]. Several studies have reported risk factors for recurrence that have not been confirmed in other series. This variation is not surprising if we just think of the very low recurrence percentages. Any result found in such low recurrence rates can be explained by the biases that may be present in every retrospective cohort study [33, 34]. We cannot claim that we confirmed indeed such factors or can propose any new ones. Indeed, the percent of tall-cell variant—which is thought to exhibit more aggressive pathologic characteristics than classic PTMCs- in our cohort was 1.5 %. All of them underwent total thyroidectomy and thyroid remnant ablation and no one had persistence or recurrence during follow-up. Of the thirteen patients with persistent disease, five of them had lymph nodes (4 CPC, 1 SVC), capsule involvement was present in three, while one of them was T > 1 and five of them N > 0. Therefore, we cannot conclude any effect of subtype, lymph node metastases or capsule involvement on outcome. The percentage of patients with persistent disease—small but not negligible—underscores the need for close follow up in all PTMC patients.

No change was noted in the mean age at diagnosis or the female to male ratio during the two time periods of the study. Furthermore, we did not find any remarkable differences in the presentation, clinical characteristics, histology and outcome of the most recently detected cases compared to those detected in previous decades. In regard to PTMC subtypes, there was a decrease in the percentage of the CPC and an increase in the percentage of N-EFVC during the latest study period. This finding is in agreement with a recent study from US where these changes in tumor histology after 2000 were accompanied by an increase in BRAF and RAS mutations, suggesting new etiologic factors [35]. Furthermore, multifocality was more often present in patients in the TP2. This may be due to the application of better and more sophisticated pathology techniques. Overall the character of the disease does not seem to have changed remarkably during these 30 years in Greece, despite the change from an iodine-deficient to an iodine-sufficient status [4].

In our series 81.8 % of the patients were diagnosed with PTMC undergoing thyroidectomy for non-malignant thyroid disease which is in agreement with previous studies in Greece that reported an even higher relative frequency of incidentally discovered PTMCs (88 %) [21, 36]. Prevalence of incidental PTMC was high but similar in the two time periods. Three decades ago, an incidence of 1 % was reported for incidental PTMCs [21] and increased about seven times in a more recent study [37]. This may be partly related to broader application of total or near total thyroidectomy in recent decades and it was reconfirmed in our study. Many other studies from other countries as well support that PTMCs are mostly incidental findings in surgeries for other pathologies non-related to thyroid malignancy [38, 39].

A recent meta-analysis [40] which included 3523 subjects (854 patients with incidental PTMC and 2669 patients with non-incidental PTMC) with mean follow-up of 70 months showed that non-incidental PTMC has different clinical characteristics (larger tumor size, higher incidence of lymph node metastases) and a much higher recurrence rate than incidental PTMCs (OR = 14.7, 95 % CI 5.6–54.8, p < 0.001). We have to mention the significant heterogeneity detected for all parameters especially in the non-incidental group. But even after excluding two studies to improve heterogeneity, recurrence rate remained much higher for non-incidental PTMCs (OR = 9.2, 95 % CI 3.5–34.42, p < 0.001) [39]. In our cohort patients with non-incidental PTMCs presented in statistically significantly higher percentages with a single nodule, loco-regional extension and/or lymph-node, capsule involvement and had higher frequency of the invasive subtype. In a significantly higher proportion, patients with non-incidental PTMCS underwent adjuvant radioiodine ablation. Nevertheless disease recurrence was observed in patients with incidental PTMCs, while persistent disease was more frequent in patients with non-incidental PTMCs but without achieving statistical significance.

In our study an important number of patients presented with hyperthyroidism. Specifically, more patients underwent thyroidectomy for toxic multinodular disease and less for Graves’ disease during time period 1 compared to patients in the time period 2. This could be explained as patients with symptoms seek for medical help visit doctors earlier. Another possible reason may be that these patients were more likely to undergo surgery especially in patients with Graves’ disease and recurrence or coexisting with single nodules. Despite the reported more aggressive course of PTC in Graves’, this was not the case for PMTC in our cohort [41–43]. Hashimoto’s thyroiditis was also present at a high percentage in patients with PTMCs in agreement with previous studies [20, 41–43]. Patients with Hashimoto’s are more closely followed up and this can explain the fact that the rate of detection of PTCs in general may be 1.5–2 times higher in these patients [21].

The advantages of our study are the large number of patients, the common origin of the patients and mainly the long term and detailed follow up. To our knowledge, this is the first study where patients were followed for at least 5 years and a mean of 10.6 years. Nevertheless the very low recurrence rate in our cohort, underscores the need for even larger sample sizes in order to ensure this non-aggressive behavior of PTMCs and justify a more conservative approach.

In conclusion, PTMCs appear to have a benign course in the majority of patients initially treated with total or near-total thyroidectomy without differences in recurrence or persistence between the radioiodine ablated and non-ablated patients. We did not note any important changes regarding clinical presentation, tumor characteristics, recurrence or persistence of PTMCs during the last three decades.

## Abbreviations

PTMC: papillary thyroid microcarcinoma
TP: time period
US: ultrasonography
TG: thyroglobulin
TSH: thyroid stimulating hormone
SD: standard deviation
CPC: classic papillary
EFVC: encapsulated follicular variant
N-EFVC: non-encapsulated follicular variant
SVC: sclerosing variant
T-CVC: tall-cell variant
